# Investigation on the genetic-inconsistent paternity cases using the MiSeq FGx system

**DOI:** 10.1080/20961790.2021.2009631

**Published:** 2022-03-16

**Authors:** Anqi Chen, Ruiyang Tao, Chengtao Li, Suhua Zhang

**Affiliations:** a Shanghai Key Laboratory of Forensic Medicine, Shanghai Forensic Service Platform, Academy of Forensic Science, Ministry of Justice, Shanghai, China; b Department of Forensic Medicine, School of Basic Medical Sciences, Shanghai Medical College, Fudan University, Shanghai, China

**Keywords:** Forensic sciences, forensic genetics, paternity testing, next generation sequencing (NGS), ForenSeq^TM^ DNA Signature Prep Kit

## Abstract

Mutations might challenge the paternity index calculation in forensic identification. While many studies have focussed on the autosomal short tandem repeats (A-STR), the mutation status of sex chromosomes and single nucleotide polymorphism (SNP) remain blank. Next generation sequencing (NGS), known as high throughput and large sequence polymorphism, is a promising tool for forensic genetics. To describe the mutation landscapes in the paternity cases with genetic inconsistencies, a total of 63 parentage confirmed paternity cases contained at least one mismatched locus have been collected. The mutations were subsequently evaluated using Verogen’s MPS ForenSeq^TM^ DNA Signature Kit and a microsatellite instability (MSI) detection kit. The result showed 98.41% (62/63) of the cases had no additional autosomal mutations even when the number of A-STRs increased to 27. As for the sex chromosomes, about 11.11% (7/63) of the cases exhibited either X-STR or Y-STR mutations. D2S1338, FGA and Penta E were the most frequent altered STRs, which suggested they might be the mutation hotspots. In addition, a male with sex chromosome abnormality was observed accidently, whose genotype might be 47, XXY, rather than MSI. Nearly 56.90% of the STR loci possessed isoalleles, which might result in higher STR polymorphisms. No Mendelian incompatibility was detected among the SNP markers, which indicated that SNP was a more reliable genetic marker in the genetic-inconsistent paternity cases.

Supplemental data for this article is available online at https://doi.org/10.1080/20961790.2021.2009631 .

## Introduction

The short tandem repeat (STR) typing method using the conventional capillary electrophoresis (CE) is the most popular fragment size analysis in the field of forensic genetics [1]. However, the method is born with limitations. High mutation rates of STRs might result in an ambiguous assignment of paternity [2]. In the past few years, many studies have investigated the STR mutations in paternity cases, and most of them emphasized either on the population data or the locus-specific mutation rates [3–6].

With the development and popularisation of next generation sequencing (NGS), more and more scientific problems had been reconsidered [7]. At the same time, many commercial kits have also been developed for forensic DNA profiling [8–11]. NGS provides the sequence information of the markers, which further improves statistical calculation and resolution for the paternity testing [12]. Li *et al*. [13] investigated the paternity testing with mismatched STR loci using the Ion Torrent Personal Genome Machine (PGM). Although the number of unique alleles increased a lot, the study did only focus on the mutations of autosomal STRs (A-STRs) with limited sample size. Up to now, the mutation status of STRs on chromosome X (X-STRs), chromosome Y (Y-STRs) and the single nucleotide polymorphism (SNP) markers have remained unknown.

Previous studies [10, 11, 14, 15] have demonstrated that Verogen’s ForenSeq^TM^ DNA Signature Prep Kit (DNA Primer Mix A) is a reliable tool for forensic DNA typing. The panel consists of 27 A-STRs, 7 X-STRs, 24 Y-STRs and 94 identity SNPs markers in a single reaction [11, 15]. The large-scale panel covers the frequently-used forensic markers, and it might provide an overview of the mutation status. Similarly, microsatellite instability (MSI) has been regarded as the “gold standard” for hypermutability evaluation in clinic practice [16]. Therefore, it might also reflect the genetic instability in the genetic-inconsistent paternity cases.

In order to investigate the mutation landscape of the locus-inconsistent paternity cases, we collected 63 paternity cases from the routine caseworks. All samples had been sequenced using the MiSeq FGx Sequencing System, and the data were analysed based on the parent-offspring relationship.

## Materials and methods

### Sample preparation

A total of 146 blood-stain samples from the 63 paternity cases (44 duos and 19 trios) were selected for the present study. Genomic DNA was extracted from the blood-stain samples, using the QIAamp DNA Investigator Kit (QIAGEN, Hilden, Germany), and quantified with a Qubit fluorometer (Life Technologies, Carlsbad, CA, USA). Extracted DNA was stored at −80 °C until use. The parent-offspring relationships in those families were confirmed using CE-based autosomal STR typing with combined paternity index (CPI) >10 000 according to the technical specifications for paternity testing issued by the Ministry of Justice of the People’s Republic of China [17], and all mismatched loci were considered as mutations. The samples were collected with the approval of the Ethics Committee of Academy of Forensic Science, Ministry of Justice, China. All participants provided written informed consent.

### CE-based genotyping

CE data of STRs were generated using the SiFaSTR^TM^ 23-plex Kit (Academy of Forensic Science, Shanghai, China). To assess the genotypes of the X-STRs and Y-STRs, the DNA was also amplified with Goldeneye® 17X Kit (Peoplespot, Beijing, China) and Goldeneye^®^ Y-Plus Kit (Peoplespot). Fluorescent multiplex polymerase chain reactions (PCR) were conducted following manufacturer’s instructions. The STRs were genotyped using a 3130xl ABI Prism Genetic Analyzer (Applied Biosystems, Bedford, MA, USA), and the resulting data were analysed using GeneMapper Software (Applied Biosystems).

### MSI stability detection

MSI status were evaluated using an MSI Detection Kit (Microread, Beijing, China), which consisted of six quasimonomorphic mononucleotide markers (BAT-25, BAT-26, NR-21, NR-22, NR-24, and MONO-27). All amplifications were conducted in accordance with the manufacturer’s protocol. Genotyping was performed in a 3130xl ABI Prism Genetic Analyzer (Applied Biosystems) by using GeneMapper Software (Applied Biosystems). The MSI was evaluated in accordance with the manufacturer’s recommendations by the size shifts in certain PCR bands.

### Library preparation, sequencing and data analysis

A total of 1 ng extracted DNA was used for the library construction using the Primer Mix A of Verogen’s ForenSeq^TM^ DNA Signature Prep Kit (Verogen, San Diego, CA, USA). The libraries were subsequently pooled and sequenced using the Miseq FGx^TM^ reagent cartridge (Verogen) according to the manufacturer’s protocols. Raw data were automatically processed using Verogen’s Universal Analysis Software (USA) at the default analysis thresholds.

## Results and discussion

In the present study, a total of 63 parentage confirmed paternity cases (19 trios and 44 duos) were collected. Coincidently, all of them possessed only one STR mutation by using the conventional CE-based genotyping. All CE-based genetic-inconsistencies were confirmed by using the NGS platform, which again confirmed the accuracy of the ForenSeq^TM^ DNA Signature system.

**Figure 1.**
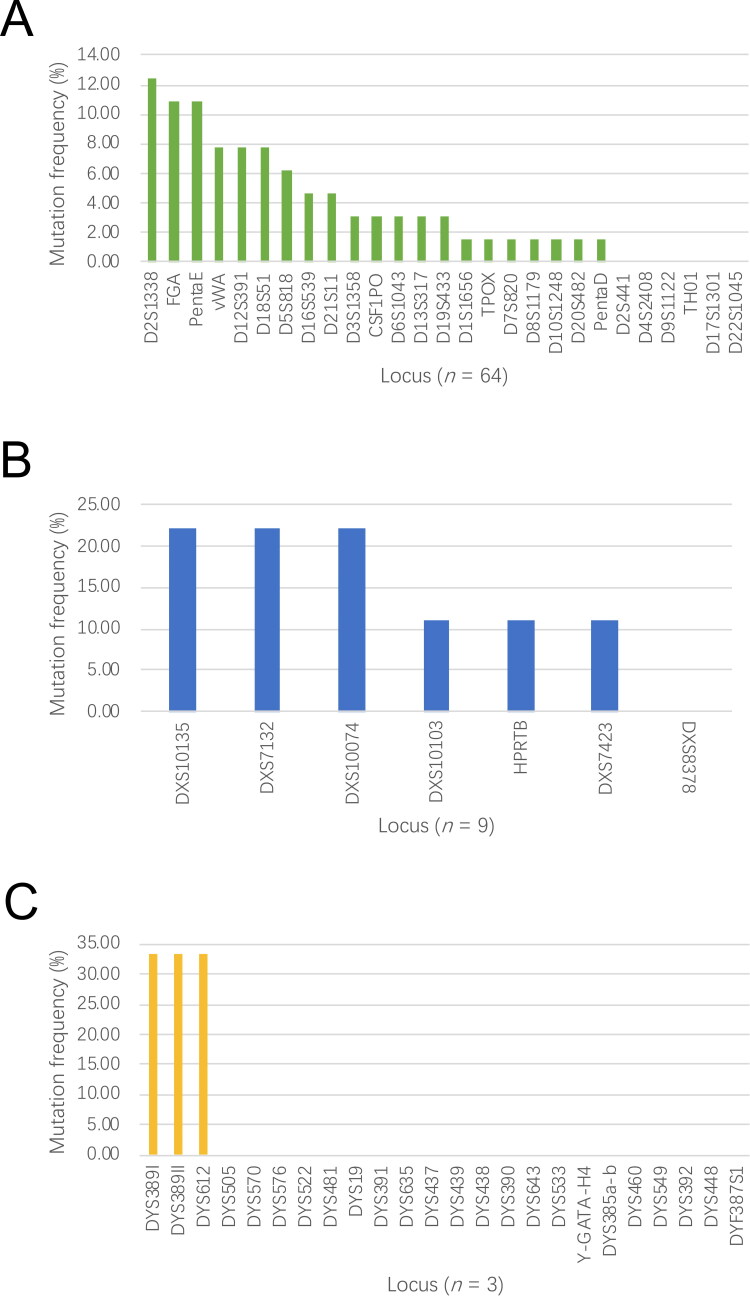
Percentage of the STR mutations in (A) A-STRs, (B) X-STRs and (C) Y-STRs.

There were 76 STR mutations detected in the study, and most of them were the A-STR mutations (84.21%), followed by X-STR mutations (11.84%) and Y-STR mutations (3.95%). The top three altered A-STRs were D2S1338 (12.50%), FGA (10.94%) and Penta E (10.94%) respectively. The most frequently mutated X-STRs were DXS10135 (22.22%), DXS7132 (22.22%) and DXS10074 (22.22%) (Figure 1). The routine paternity testing in our laboratory consists of 21 autosomal STR loci and a sex-related polymorphism Amelogenin (Amel). The NGS application accessed to a wide range of genetic markers, therefore, it should possess more chances to find a mutation. Nevertheless, only one case (Case 9) showed an additional A-STR mutation (D20S482), which suggested that the autosomal mutation was not frequent in human. The observation was in concordant with the result released by Li *et al.* [13]. As for the mutation status of X-STRs and Y-STRs, there were nine X-STR mutations (Case 18, 25, 42 and 46) and three Y-STR mutations (Case 52 and 53) being found out by NGS (Supplementary material 1).

STRs are known as microsatellite (MS) or simple sequence repeats (SSRs) [18]. The three- to four- repeated nucleotides have been frequently used in forensic genetics for personal identification [19, 20], and the one- to two- repeated nucleotides are widely used in clinic practice for hypermutability evaluation [18]. Previous studies have demonstrated that MSI is an effective tool for genetic instability evaluation [21, 22], and Case 46 might be a candidate of genetic instability due to the extremely frequent altered X-STRs. To verify the hypothesis, we assessed the MSI status of all cases. However, no MSI positive signal had been observed (data not shown), which suggested that the genetic hypermutability did not occur in the genetic-inconsistent paternity cases, even in Case 46.

**Figure 2.**
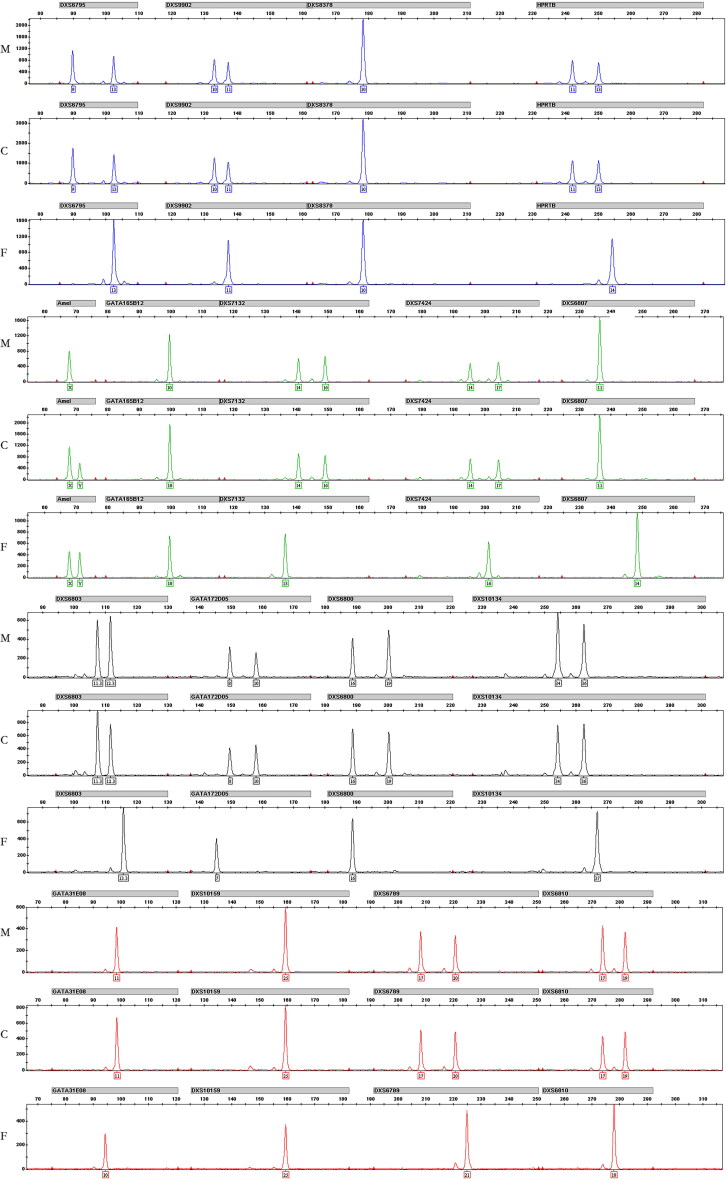
Electropherogram of genotypes at the 17X STRs (Case 46). F: father; M: mother; C: child.

Apparently, the hypothesis of genetic hypermutability could not explain the abnormal X-STR mutations in Case 46. As shown in Supplementary material 1, there were extra copies observed in each of the X-STRs. Moreover, the ratio between the total reads of X-STRs and A-STRs were 0.40 (mother), 0.44 (child) and 0.24 (father) respectively (Table 1). The values in the mother and the child were approximately two times of that in the father, which suggested that the child might have two chromosome X. As we all know, patients with Klinefelter’s syndrome (KS) is defined by 47,XXY karyotype [23, 24]. Since all the cases were parentage confirmed, we speculated the mutations might not result from the random DNA replication error but the chromosome disorder. To further authenticate the conjecture, we genotyped the 17 X-STR loci of Case 46. As expected, the result confirmed the child possessing two copies of chromosome X. In addition, all X-STR loci were exactly the same with the mother, which suggested the extra X chromosome might come from the mother (Figure 2). Unfortunately, the karyotype test was not applicable due to the sample limitations. Nevertheless, the reads of Case 46 were in concordance with the theoretical chromosomal condition (Table 1). The result suggested that Verogen’s ForenSeq^TM^ DNA Signature Prep Kit not only played important roles in personal identification, but also in chromosome abnormality screening.

**Table 1.** Summary of the sequence reads of Case 46.

Loci	Mother	Child (son)	Father
A-STRs and Amel	52913	31054	37637
X-STRs	21292	13537	8906
Y-STRs	NA	20189	24401
Ratio of X-STRs:A-STRs	0.402396387	0.435918078	0.236628849

NA: not applicable.

The CE-based STR genotyping distinguishes the alleles by fragment length, which ignores the identification of isoalleles [25]. In the present study, nearly half of the STRs (51.56%, 33 out of 64) possessed isoalleles. The 146 samples produced a total of 635 sequenced-based alleles, which was 183 alleles more than the length-based alleles (Table 2). The identification of those sequence-based alleles made the loci more polymorphic, which would subsequently improve allelic diversity and discriminatory. Moreover, the origin of mutation could be clearly interfered in the paternity cases with the improvements of sequence information. For example, the length-based genotype at D2S1338 of the child and the father were (18,24) and (23,23) in Case 7 (duo). In theory, the mutation should be either allele 18 by loss of five repeats or allele 24 by gain of one repeat. The sequence structure of the father’s allele was [TGCC]_7_[TTCC]_13_[GTCC][TTCC]_2_ (allele 23), which was different from that of [TGCC]_7_[TTCC]_11_ (allele 18, child). Therefore, the allele 24 ([TGCC]_7_[TTCC]_14_[GTCC][TTCC]_2_) might be inherited from the father by gain of one repeat. Although the past experience also suggested that most of the mutations were likely one-step mutation [26], the sequence provided more direct evidence for the empiricism. In Case 57 (trio), the sequence structure of the allele 21 (child) was different from that of the father, and we could infer that it might be inherited from the mother’s allele [AGAT]_13_[AGAC]_9_ by loss of one repeat. In general, all mutations observed in the present study were one-step mutations (Supplementary material 1), which was in concordant to the data released by Xu *et al.* [3]. The sequence structures of the X-STRs in Case 46 were exactly the same with the mother, which again interfered that it was the mother who gave two copies of chromosome X to the child (Supplementary material 1).

**Table 2.** Number of unique alleles from the 146 samples.

Locus	Number of alleles		
	Length-based	Sequence-based	Difference
CSF1PO	7	8	1
D10S1248	8	8	0
D12S391	12	36	24
D13S317	8	16	8
D16S539	6	6	0
D17S1301	8	9	1
D18S51	13	14	1
D19S433	11	11	0
D1S1656	11	14	3
D20S482	7	8	1
D21S11	13	32	19
D22S1045	9	10	1
D2S1338	11	28	17
D2S441	9	12	3
D3S1358	6	11	5
D4S2408	5	7	2
D5S818	9	13	4
D6S1043	14	14	0
D7S820	9	10	1
D8S1179	8	17	9
D9S1122	7	12	5
FGA	14	14	0
PentaD	11	11	0
PentaE	19	19	0
TH01	7	7	0
TPOX	5	5	0
vWA	8	12	4
DXS10074	10	11	1
DXS10103	7	9	2
DXS10135	17	32	15
DXS7132	8	8	0
DXS7423	5	5	0
DXS8378	6	6	0
HPRTB	6	6	0
DYF387S1	8	28	20
DYS19	5	5	0
DYS385a-b	10	12	2
DYS389I	4	4	0
DYS389II	6	19	13
DYS390	5	10	5
DYS391	4	4	0
DYS392	5	5	0
DYS437	3	5	2
DYS438	4	5	1
DYS439	4	4	0
DYS448	6	11	5
DYS460	4	4	0
DYS481	7	7	0
DYS505	6	6	0
DYS522	6	6	0
DYS533	4	4	0
DYS549	4	4	0
DYS570	9	9	0
DYS576	7	7	0
DYS612	11	15	4
DYS635	6	10	4
DYS643	5	5	0
Y-GATA-H4	5	5	0
Number of A-STRs	255	364	109
Number of X-STRs	59	77	18
Number of Y-STRs	138	194	56
Total	452	635	183

The character of high-throughput is one of the most important advantages of NGS, which made it possible to test numerous markers in parallel. Despite of the 27 STRs, there were 94 identity SNP markers included in the panel. It is known that there are many millions of potential SNPs in human genomes [27, 28], and SNP has been regarded as an alternative marker for forensic identification because of the low mutation rate [9, 28, 29]. In the present study, although there were different kinds of STR mutations in the cases, not one SNP mutation had been observed (data not shown). The result suggested that the STR mutation did not accompany with the occurrence of SNP mutation. In addition, SNPs were much more stable than STRs, even in the genetic-inconsistent paternity cases.

## Conclusion

More STR polymorphisms had been distinguished by using the NGS since the full sequences were detected. Improved polymorphisms might subsequently increase the discrimination power, which might benefit forensic identifications. Besides, the sequence information also helped recognise the origin of the mutation in some of the cases. Large-scale panel of Verogen’s ForenSeq^TM^ DNA Signature Prep Kit not only benefitted personal genotyping in the field of forensic science, but also assisted to screen the chromosomal abnormalities in the field of clinic practice. The SNP markers were more stable than the STR markers, and none of the SNP mutation did occur even in the genetic-inconsistent paternity cases. In general, abundant information provided by NGS might help construct a deeper and more comprehensive understanding in the paternity cases with mismatched STR loci.
